# Commentary: Efficacy and safety of vonoprazan-amoxicillin dual therapy versus bismuth-containing quadruple therapy for patients with *Helicobacter pylori* infection: a meta-analysis

**DOI:** 10.3389/fmicb.2025.1733070

**Published:** 2025-12-10

**Authors:** Alejandro Chen Liang, Yeison Cruz Castillo, Salma Alejandra Beltrán Covarrubias

**Affiliations:** 1 Centro de Estudios Universitarios Xochicalco Campus Tijuana, Tijuana, Mexico; 2 Universidad Autónoma de Santo Domingo, Santo Domingo, Dominica; 3 Universidad de Guadalajara, Guadalajara, Mexico; 4 Centro Universitario de Ciencias de la Salud, Guadalajara, Mexico

**Keywords:** PCAB, PPI, *H. pylori* (*Helicobacter pylori*), vonoprazan, bismuth-based quadruple therapy (BQT)

We read with interest the meta-analysis by Li et al. (2025), comparing vonoprazan–amoxicillin (VA) dual therapy with bismuth-based therapy (BQT) for *Helicobacter pylori* (*H. pylori*) eradication. We congratulate the authors on their timely and well-conducted study addressing a clinically relevant question in *H. pylori* management, which was designed to compare the efficacy and safety of VA dual therapy with BQT across randomized controlled trials (RCT). We acknowledge the authors for their effort in performing a comprehensive systematic review and meta-analysis on an important clinical topic.

We would like to respectfully raise a methodological consideration. Some included studies had BQT arms in which vonoprazan (VPZ) was used instead of a proton pump inhibitor (PPI) in the control group, such as the RCT by Ratana-Amornpin et al. (2023), and Gaozhong and Qing (1974), Because VPZ does not require an acidic environment for activation, it provides a stronger and more sustained acid suppression, resulting in faster symptom control compared to PPIs (Agago et al., 2024). Moreover, in a meta-analysis by Simadibrata et al. (2022), comparing potassium- competitive acid blockers (PCABs), the drug class to which VPZ belongs, the H. pylori eradication rate was significantly higher with PCABs compared to PPIs. Therefore, including these studies may introduce heterogeneity, affect comparability and shadow a true effect. Notably, Table 1 in the data extraction reports a PPI rather than VPZ, which contradicts the original publication. This inconsistency may misrepresent the study and should be corrected to ensure accurate reporting.

Regarding study selection, including certain trials from the chosen databases may result in a sample that is not fully homogeneous, as some studies do not entirely meet the inclusion criteria. Highlighting these points emphasizes methodological transparency and helps strengthen the reliability and interpretability of the conclusions. Careful screening and verification of eligibility at multiple stages could have prevented such errors, underscoring the importance of rigorous selection processes. Following the PRISMA guidelines from the outset would have facilitated standardized documentation of inclusion and exclusion steps, improved reproducibility and minimizing methodological bias. This approach aligns with the recommendations of Calderon Martinez et al. (2025), in a Comprehensive Guideline to Conduct a Systematic Review and Meta-Analysis in Medical Research, which underscore the importance of using a well-defined PICO framework and carefully identifying appropriate studies to minimize bias and ensure robust evidence synthesis.

Finally, the review did not specify which types of quadruple therapy were included. In China, quadruple therapy can combine different antibiotics with either a PPI or VPZ, and it is important to clarify which combinations were considered. Providing this detail would enhance transparency and allow for a more precise comparison of the included studies. Highlighting these points aims to support methodological rigor and strengthen the reliability of the conclusions.

Our aim is to provide a constructive contribution to the discussion on optimal *H. pylori* management and to ensure the meta-analysis conclusions are based on accurate data.
